# Supplementary material for: Transplantable human thyroid organoids generated from embryonic stem cells to rescue hypothyroidism
Source: Nat Commun. 2022 Nov 17;13:7057. doi: 10.1038/s41467-022-34776-7 (PMC9672394; doi:10.1038/s41467-022-34776-7)
Supplement: Supplementary file 1 — Supplementary Information [file 41467_2022_34776_MOESM1_ESM.pdf]

Supplementary Information

Figures

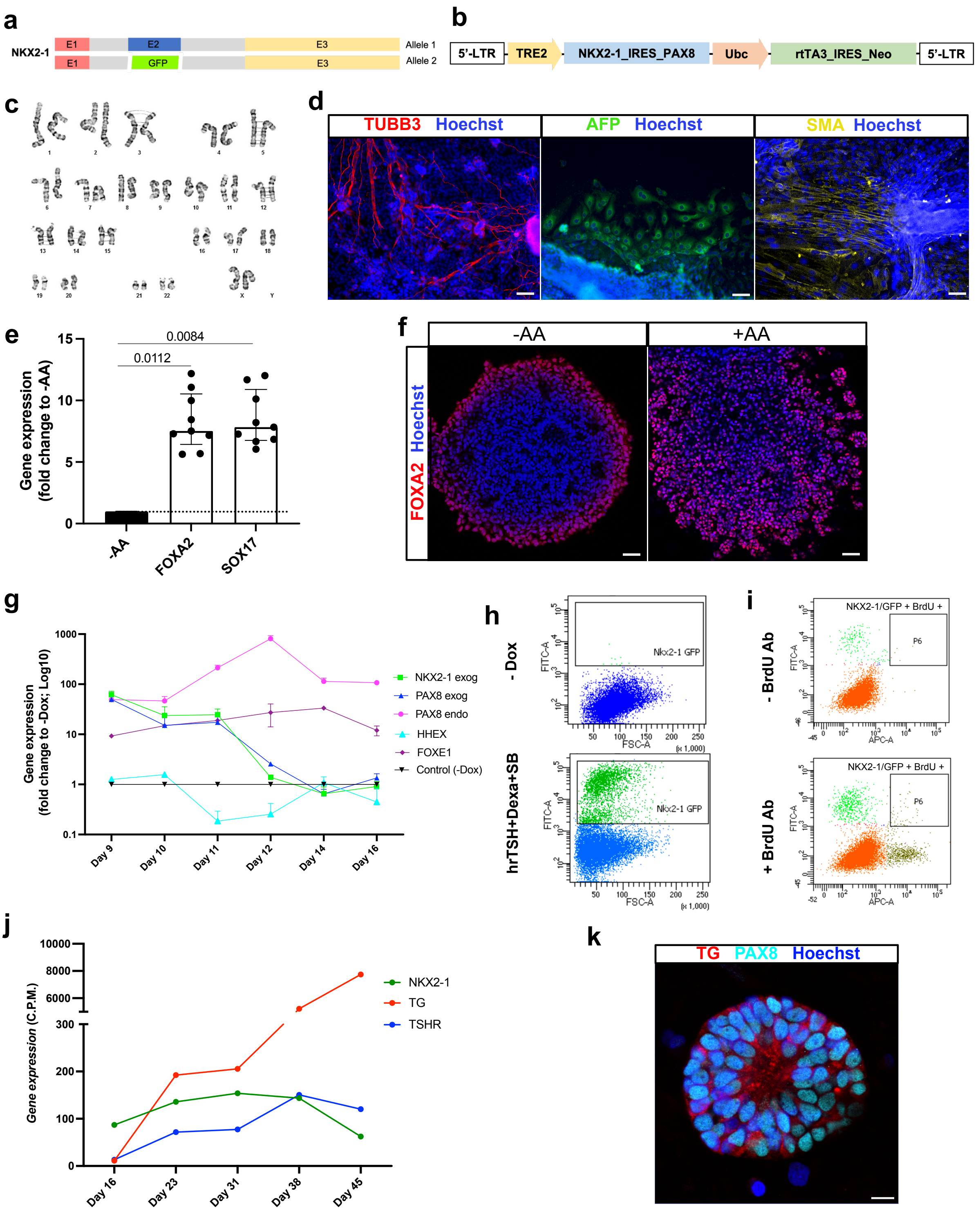

Supplementary Fig. 1 Generation, characterization and differentiation of the NKX2-1-PAX8 tetracycline-inducible human ESC line. (a) Schematic representation of the

previously generated NKX2-1$^{WT/GFP}$ human ES cell line [31]. (b) NKX2-1-PAX8 tetracycline-inducible human ESC line was generated by cloning ORFs into the pInducer20 lentivirus vector backbone. (c) Human ESCs showed normal karyotype after genetic manipulations. (d) Modified hESCs pluripotency maintenance was confirmed by spontaneous differentiation into ectoderm (TUBB3), endoderm (AFP) and mesoderm cells (αSMA). (e) *FOXA2* and *SOX17* mRNA levels after activin A (AA) treatment (day 5) (n = 9 per gene). (f) Immunostaining shows increase in the proportion of FOXA2+ cells after treatment with Activin A. (g) qRT-PCR analysis shows pattern of exogenous *NKX2-1* and *PAX8* and endogenous *PAX8, FOXE1* and *HHEX* expression from day 9 to day 16 of the differentiation protocol (n = 3 per time point). (h-i) Flow cytometry plots showing the gate strategy for the selection NKX2-1^{GFP} cell population (h; -Dox was used as negative control) and double positive NKX2-1^{GFP} /BrDU+ cells (-BrdU antibody was used as negative control) (one representative experiment at Day 58). (j) Bulk-RNAseq data showing curve of NKX2-1, TG and TSHR gene expression from day 16 to day 45 of the differentiation protocol. (k) NKX2-1 and TG co-staining, at day 28, shows beginning of follicular organization, without the monolayer epithelium, but with the appearance of the luminal compartment. Two-sided Mann-Whitney test was used for statistical analysis (p values are presented in the graph; data presented as median (IQR)). The experiment was performed three times with similar results (d, f, k). Scale bars, 50 µm (d,f) and 10µm (k). Source data are provided as a Source Data file.

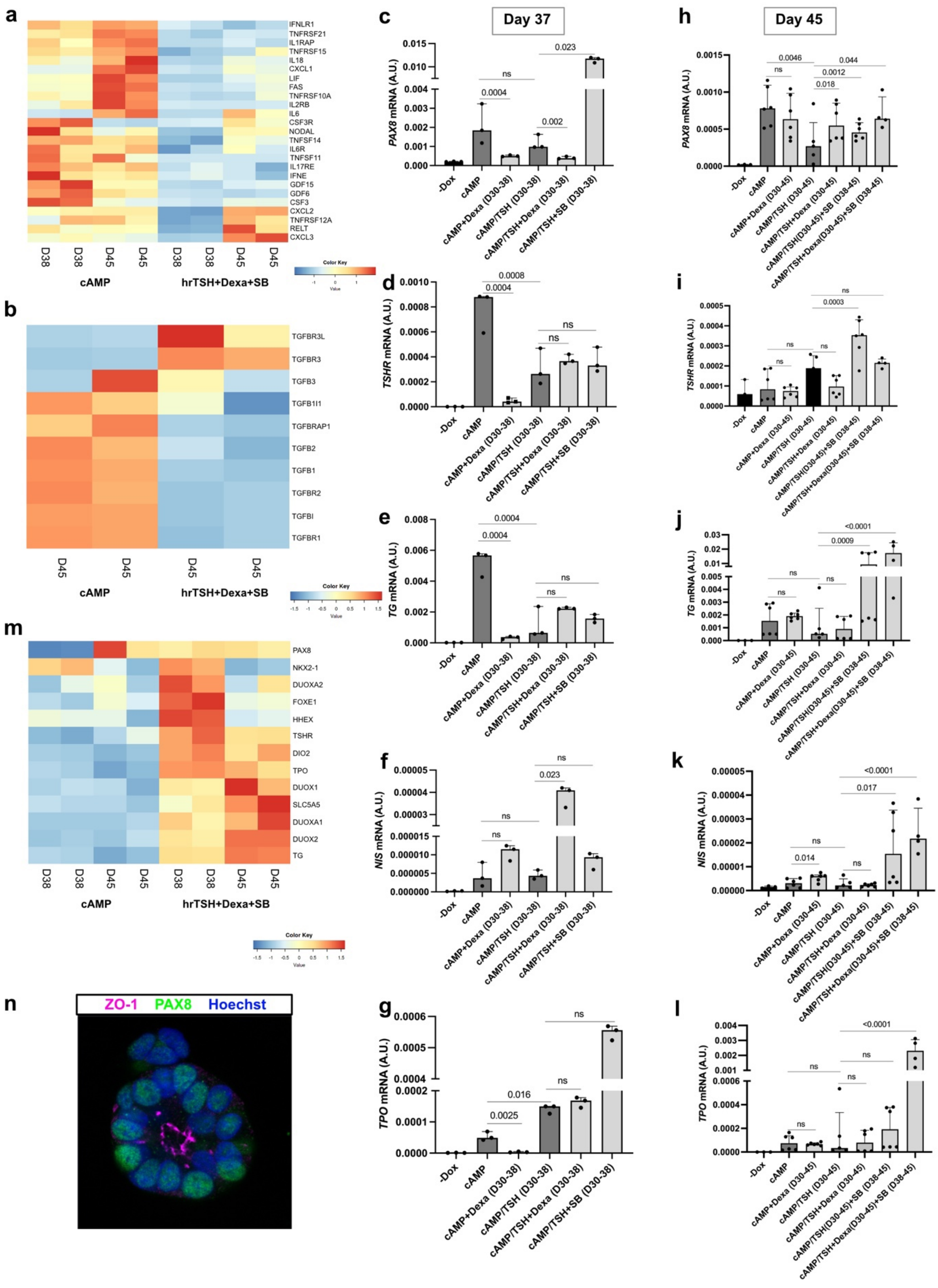

Supplementary Fig. 2 Changes of supplementation conditions and later stages of protocol definition. (a-b) Heatmap of normalized bulk RNA-Seq expression of (a) inflammation and (b) TGFβ signaling markers within NKX2-1^{GFP} cells at day 38 and 45 of the differentiation

protocol showing the effect of switching from cAMP treatment to hrTSH+Dexa+SB from day 30. (c-i) qRT-PCR analysis performed at day 37 and day 45 of differentiation protocol comparing different conditions and controls tested to establish the final protocol ("/" indicates switch from one treatment to the new one). *PAX8, TSHR, TG, NIS* and *TPO* mRNA levels show the effect of switch of cAMP condition to hrTSH supplemented with Dexa and/or SB on thyroid maturation at day 37 (c-g; n = 3) and day 45 (h-l; n = 3 for -Dox, n = 4 for cAMP/TSH+Dexa (D30-45)+SB (D38-45); n = 5 for cAMP/TSH (D30-45); n = 6 for the other conditions). (m) Heatmap of normalized bulk RNA-Seq of thyroid genes expression among NKX2-1^{GFP} cells at days 38 and 45 of the thyroid differentiation protocol comparing the expression levels within cells treated with cAMP or hrTSH+Dexa+SB. Rows represent markers and columns represent specific time points. Color values in the heatmap represent mean expression levels. (n) PAX8 and ZO-1 co-staining, at day 45, shows the single-layered thyroid follicular epithelium and the delimited lumen formed. Two-sided Mann-Whitney test was used for statistical analysis (p values are presented in the graphs; ns= not significant; data presented as median (IQR)). The experiment was performed three times with similar results (n). Scale bars, 10 μm. Source data are provided as a Source Data file.

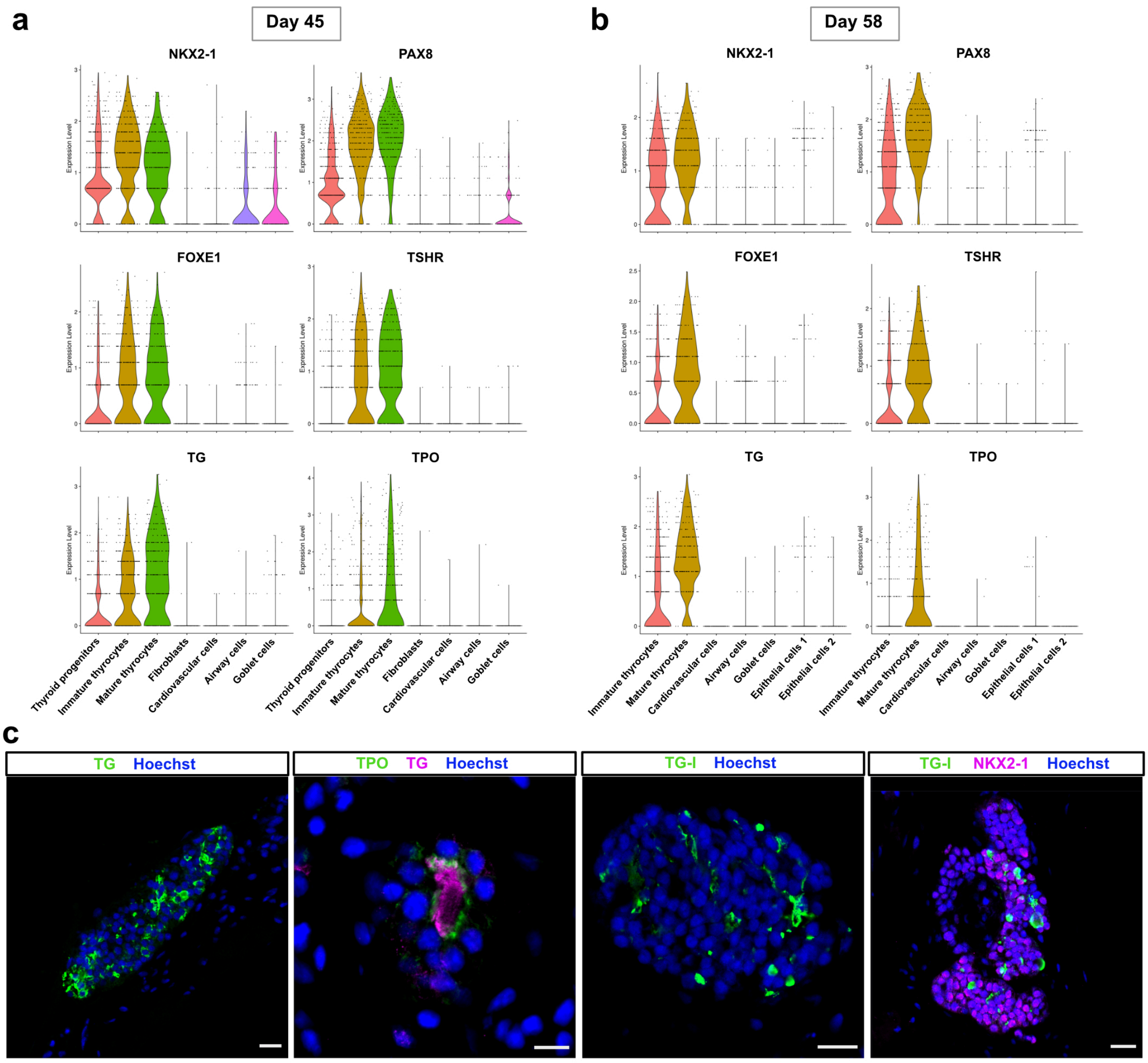

Supplementary Fig. 3 scRNA-Seq thyroid clusters characterization at day 45 and day 58 and immunostaining of organoids at day 70 . (a-b) Violin plots showing expression levels of key thyroid markers among all clusters at day 45 and day 58 of thyroid differentiation protocol. (c) Immunostaining for TG, TPO, TG-I and NKX2-1 shows the presence of follicular structures with functional properties at day 70. The experiment was performed three times with similar results (c). Scale bars, 20 µm and 10 µm (TPO).

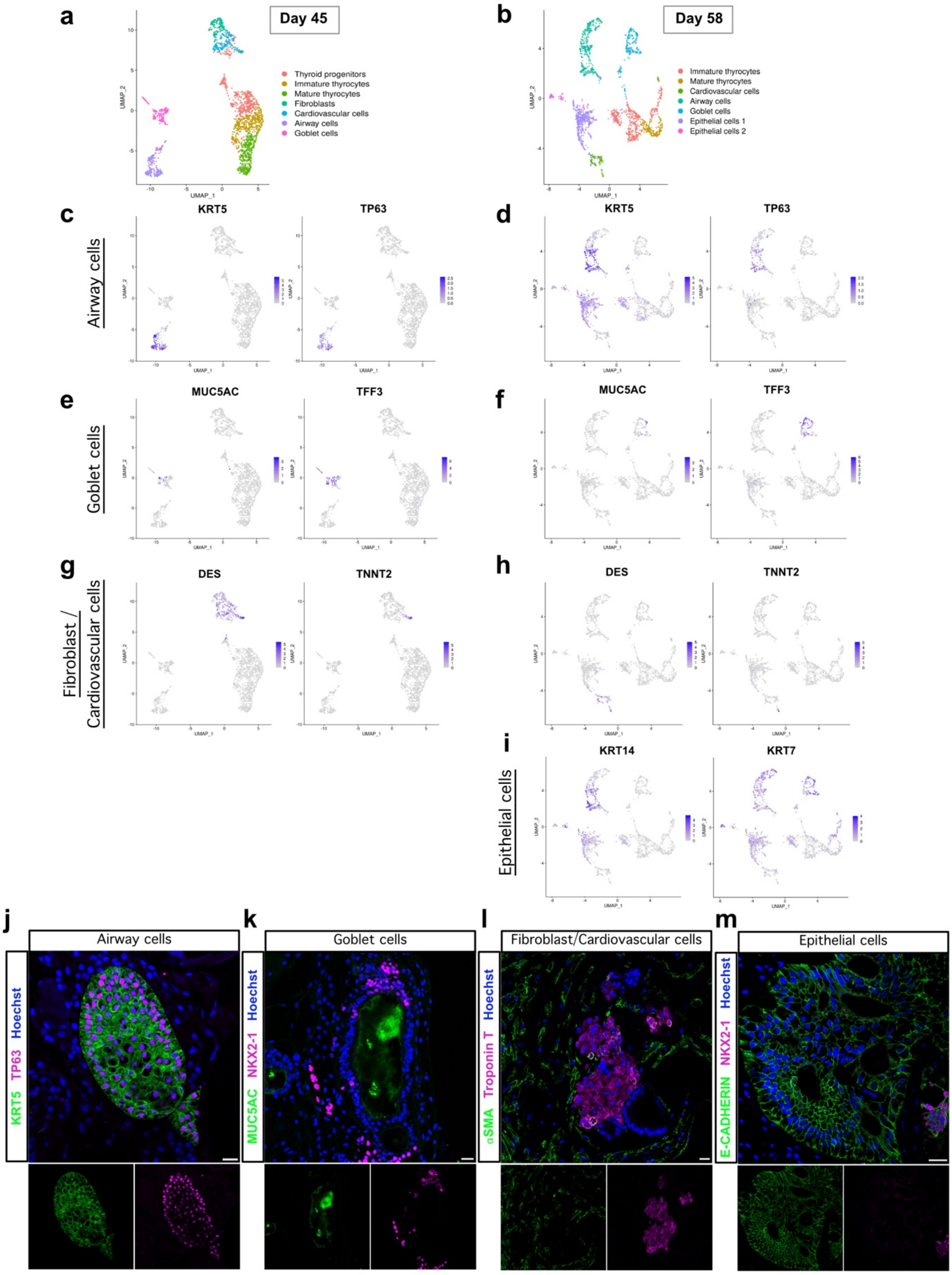

Supplementary Fig. 4 scRNA-Seq characterization of non-thyroid clusters from day 45 and day 58. (a-i) Single cell RNA-Seq unsupervised clustering of *in vitro* hESC-derived human thyroid organoid model cells at (a) day 45 and (b) day 58. Each cluster is represented

by a specific color. (c-i) UMAP plots showing the expression of differentially expressed specific markers for non-thyroidal clusters. (c-d) UMAP plots for KRT5 and TP63 genes characterize airway cluster cells; (e-f) MUC5AC and TFF3 genes characterize Goblet cells; (g-h) DES and TNNT2 genes characterize fibroblasts and cardiovascular cells, respectively; and (i) KRT14 and KRT7 genes characterize the epithelial cells 1 and 2 clusters detected at day 58. (j-m) Immunostaining characterization of non-thyroid cell types. (j) co-expression of KRT5 and TP63 characterizing airway cells (basal cells; representative image at day 58); (k) MUC5AC and NKX2-1 characterizing goblet cells (representative image at day 45); (l) αSMA and Troponin T characterizing fibroblasts and cardiovascular cells (representative image at day 45); and (m) E-Cadherin characterizing the non-thyroid epithelial cells (NKX2-1 negative; representative image at day 58). The experiment was performed three times with similar results (j-m). Scale bars, 20 µm.

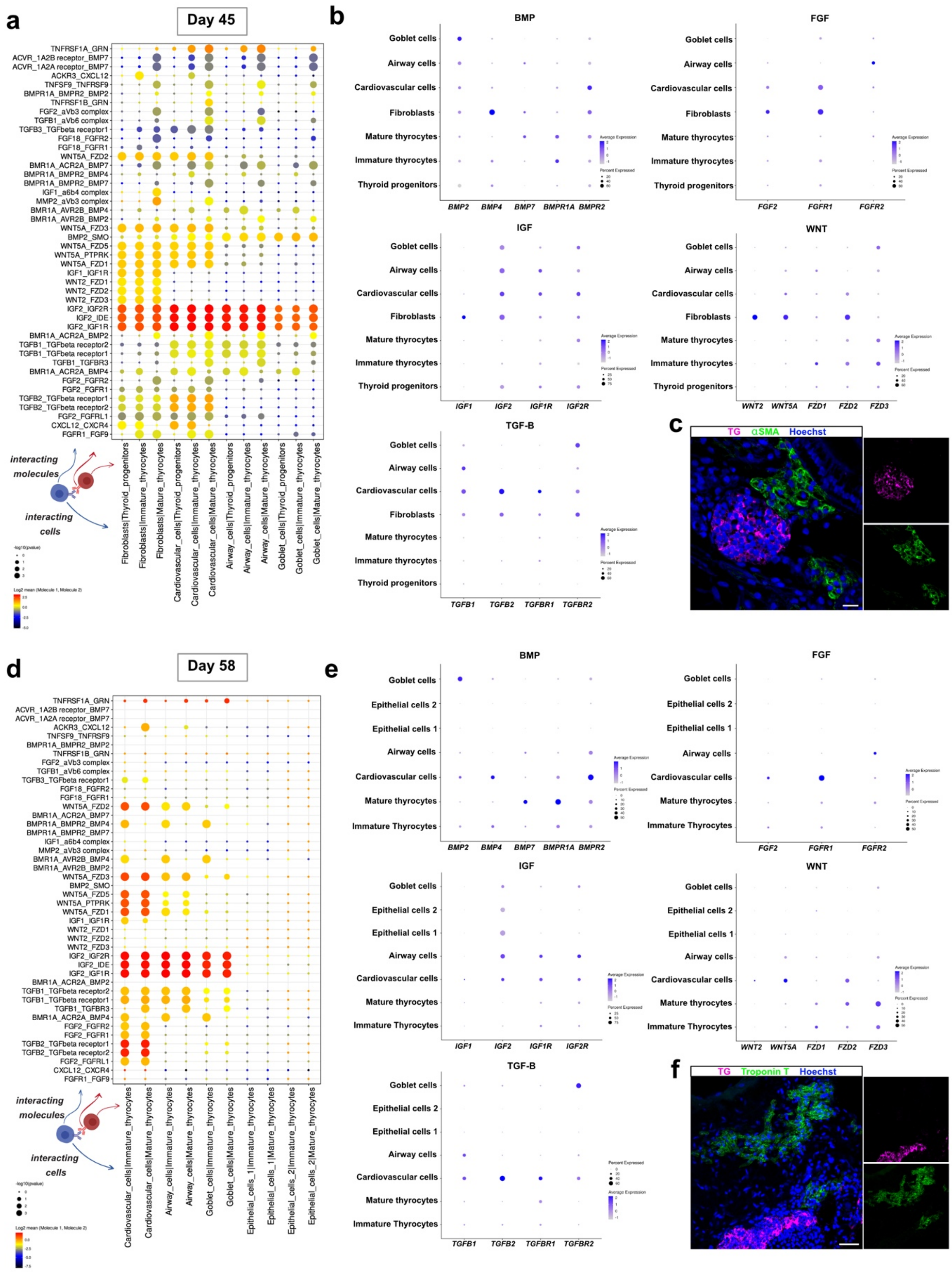

Supplementary Fig. 5 CellPhone-DB heterotypic interaction between thyroid populations and other cells at day 45 and day 58. Diagram showing selected ligand-receptor interactions using CellPhoneDB on the single-cell datasets of human thyroid organoids at (a) day 45 and

(d) day 58; P values are indicated by circle size. The color scale shows the Log2 mean values of the average expression level of interacting molecule 1 from cluster 1 and interacting molecule 2 from cluster 2. (b,e) Dot plot visualization of markers expression from selected relevant interactions data across clusters. Shown are the expression levels of receptors and ligands for BMP, FGF, IGF, WNT and TGFβ pathways. The size of the circles indicates the percentage of expression. The color scale bar indicates the mean values of the average expression levels. Immunostaining for (c) TG and αSMA and (f) TG and Troponin T shows the close interaction between thyroid cells and mesodermal cells, at day 45 and with cardiovascular cells at day 58, respectively. The experiment was performed three times with similar results (c, f). Scale bars, 20 μm.

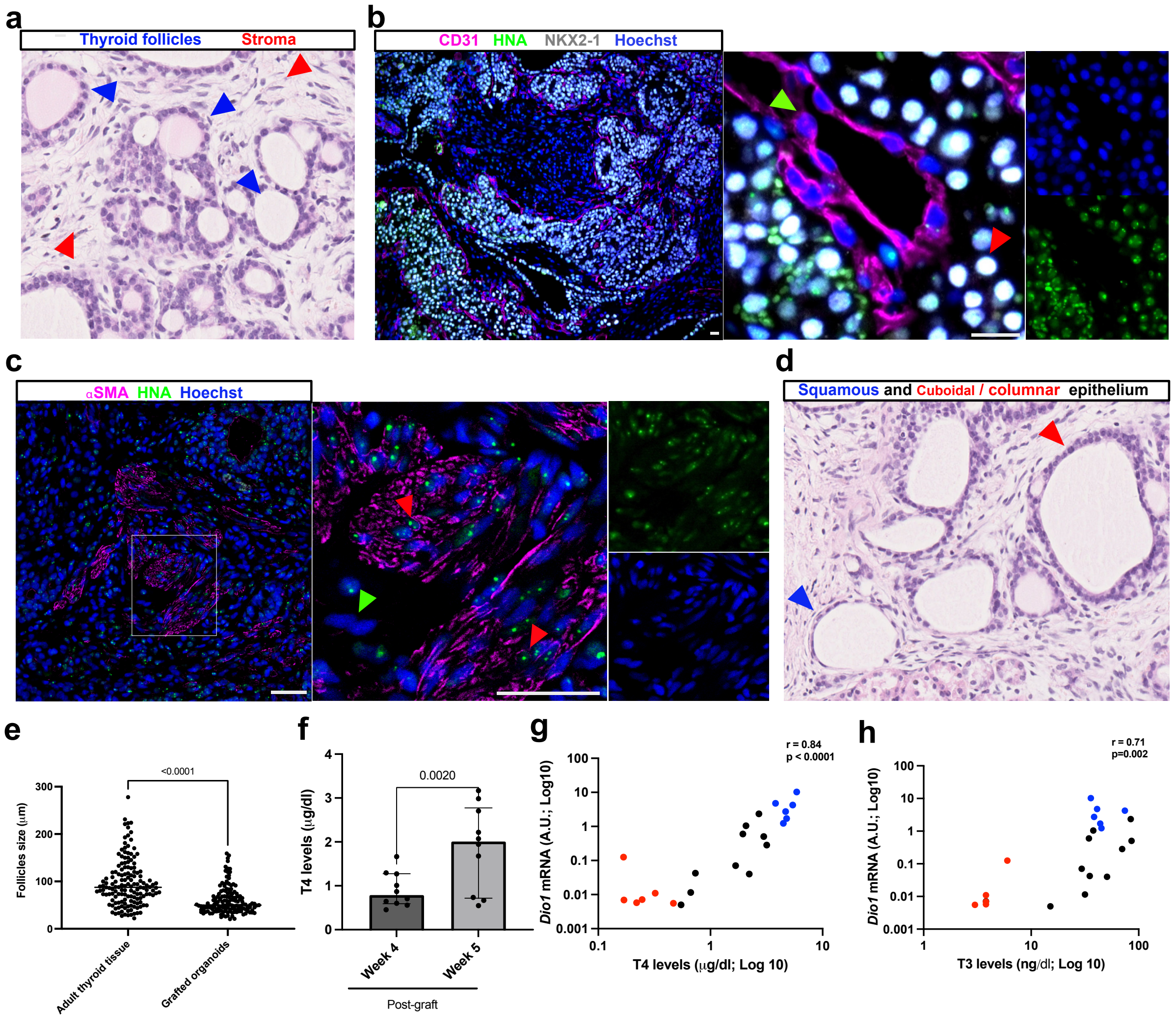

Supplementary Fig. 6 Histological characterization of the grafted organoids and analysis of the thyroid function. (a) H&E staining shows the presence of multiple thyroid follicles (blue arrows) surrounded by stromal cells (red arrows). (b) NKX2-1 and CD31 immunostaining demonstrate the presence of a dense network of small blood vessels in close proximity to the thyroid follicles. In contrast to NKX2-1+ cells (red arrow), CD31+ cells do not express HNA (green arrow), providing clear evidence that the endothelial cells are host derived. (c) Co-staining of αSMA and HNA (red arrows) indicates a human origin of the stromal cells (green arrow indicates the HNA negative cells). (d) Histological analysis shows the presence of active follicles with cuboidal/columnar epithelium (red arrows) and inactive follicles with flat/scaly cell organization (blue arrows). (e) Dot plot graph showing the size distribution of thyroid follicles within adult human thyroid tissue and hESC-derived graft tissue (n = 150 per group). (f) T4 levels among grafted mice at week 4 and 5 post-graft (n = 10 per group). (g-h) Correlation of hepatic *Dio1* mRNA with plasma (g) T4 and/or (h) T3 levels among control (blue dots), RAI-ablated (red dots) and RAI-ablated and grafted (black dots) mice. Two-sided Mann-Whitney (e-f) and Spearman Correlation (f-h) tests were used for statistical analysis (r and p values are presented in the graphs; data presented as median (IQR)). The experiment was performed using the 10 graft samples with similar results (a-d). Scale bars, 20 µm. Source data are provided as a Source Data file.

Tables

Supplementary table 1: Human differentiation medium composition

**hESC Differentiation Medium**	**Stock concentration**	**Final concentration**	**Volume (50 ml)**
DMEM/F12 + Glutamax			38.4 ml
FBS		20% v/v	10 ml
MEM-Non-Essential Amino Acids (MEM-NEAA)	100x	1%	500 µl
Sodium pyruvate	100x	1%	500 µl
P/S	100x	1%	500 µl
2-Mercaptoethanol (in PBS)	7%	0.007%	50 µl
Vitamin C	50 mg/ml		50 µl

Supplementary table 2: List of primers sequences used for qRT-PCR analysis.

**Gene name**	**Primer Forward**	**Primer Reverse**	**Specie**
FOXA2	GGGAGCGGTGAAGATGGA	TCATGTTGCTCACGGAGGAGTA	Human
SOX17	GTGGACCGCACGGAATTTG	CACGTCAGGATAGTTGCAGTAAT	Human
exNXK2-1	TGTCCTGCTCCACCTTGCT	CGCACACCGGCCTTATTCCA	Human
exPAX8	CCTCGGTGCACATGCTTTAC	GAGGTCTGCCATTCACAAAGG	Human
PAX8	CGAGCGACTCCCCGGCGAT	GAGGTCTGCCATTCACAA	Human
FOXE1	GCGACAACCCCAAAAAGTGG	GCCCAGTAGCCCTTACC	Human
HHEX	GGACGGTGAACGACTACACGCA	CCAGACGCTTCCTCTCGGCGC	Human
TG	AGACACCTCCTACCTCCCTCA	TCCTTGGACATCGCTTTGGC	Human
TSHR	TGACCTTTCTTACCCAAGCCA	TGCTCTCAAGGACTTACACATCA	Human
TPO	CTGTCACGCTGGTTATGGC	GCTAGAGACACGAGACTCCTCA	Human
NIS/SLC5A5	ATCGCTATGGCCTCAAGTTCC	TCCAGGTACTCGTAGGTGCT	Human

GAPDH	GTCTCCTCTGACTTCAACAGCG	ACCACCCTGTTGCTGTAGCCAA	Human
Dio1	ATGGCCAGGAACCCCCG	GCCTGCTGCCTTGAATGAAA	Mouse
B2microglobulin	GCTTCAGTCGTCAGC ATGG	CAGTTCAGTATGTTCGGCTTCC	Mouse

Supplementary table 3: List of primary and secondary antibodies used in the experiments.

**Primary antibodies**					
Protein Target	***Provider***	***Catalog number***	***Host Species***	***Dilution Flow Cyt.***	***Dilution IF***
AFP	Santa Cruz	sc-8108	Goat		1:100
β-III Tubulin	Eurogentec	MMS-435P-200	Mouse		1:1,000
αSMA	Abcam	ab32575	Rabbit		1:1,000
NKX2-1	Abcam	ab76013	Rabbit		1:500
PAX8	Cell Signaling	59019	Rabbit	1 :100	1:500
TG	Dako	A0251	Rabbit		1:2,000
TG	Abcam	Ab187378	Mouse		1:250
TPO	Santa Cruz	sc-58432	Mouse		1:100
T4	Biorbyt	orb11479	Goat		1:1,000
T4	Invitrogen	MA5-14716	Mouse		1:100
TG-I	Gift from C. Ris-Stalpers		Mouse		1:250
E-cadherin	BD	610181	Mouse		1:1,000
CD31	R&D	AF3628	Goat		1:100
HNA	Abcam	ab190710	Mouse		1:250
Phalloidin 647	Invitrogen	10656353			1:100
FOXA2	Abcam	Ab40874	Rabbit		1:1,000
SOX17	Santa Cruz	Sc-17355	Goat		1:100

Troponin T	Abcam	Ab8295	Mouse		1:200
MUC5AC	Abcam	Ab3649	Mouse		1:250
TP63	Abcam	Ab735	Mouse		1:50
KRT5	Cell Signaling	#25807	Rabbit		1:500
**Secondary antibodies**					
Cy3-conjugated	Jackson Immunoresearch	715-165-150	Donkey anti-mouse IgG		1:500
Cy3-conjugated	Jackson Immunoresearch	711-165-152	Donkey anti-rabbit IgG		1:500
Cy3-conjugated	Jackson Immunoresearch	705-165-147	Donkey anti-goat IgG		1:500
Alexa fluor 488-conjugated	Jackson Immunoresearch	715-545-150	Donkey anti-mouse IgG		1:500
Alexa fluor 647-conjugated	Jackson Immunoresearch	715-605-150	Donkey anti-mouse IgG		1:500
Alexa fluor 647-conjugated	Jackson Immunoresearch	711-605-152	Donkey anti-rabbit IgG	1:300	1:500
