# Supplementary material for: Transplantable human thyroid organoids generated from embryonic stem cells to rescue hypothyroidism
Source: Nat Commun. 2022 Nov 17;13:7057. doi: 10.1038/s41467-022-34776-7 (PMC9672394; doi:10.1038/s41467-022-34776-7)
Supplement: Supplementary file 3 — Reporting Summary [file 41467_2022_34776_MOESM3_ESM.pdf]

Corresponding author(s): Sabine Costagliola
Last updated by author(s): Oct 24, 2022

Reporting Summary

Nature Portfolio wishes to improve the reproducibility of the work that we publish. This form provides structure for consistency and transparency in reporting. For further information on Nature Portfolio policies, see our Editorial Policies and the Editorial Policy Checklist.

Statistics

For all statistical analyses, confirm that the following items are present in the figure legend, table legend, main text, or Methods section.

n/a	Confirmed	
☐	☒	The exact sample size (*n*) for each experimental group/condition, given as a discrete number and unit of measurement
☐	☒	A statement on whether measurements were taken from distinct samples or whether the same sample was measured repeatedly
☐	☒	The statistical test(s) used AND whether they are one- or two-sided<br>*Only common tests should be described solely by name; describe more complex techniques in the Methods section.*
☒	☐	A description of all covariates tested
☐	☒	A description of any assumptions or corrections, such as tests of normality and adjustment for multiple comparisons
☐	☒	A full description of the statistical parameters including central tendency (e.g. means) or other basic estimates (e.g. regression coefficient) AND variation (e.g. standard deviation) or associated estimates of uncertainty (e.g. confidence intervals)
☐	☒	For null hypothesis testing, the test statistic (e.g. *F*, *t*, *r*) with confidence intervals, effect sizes, degrees of freedom and *P* value noted<br>*Give P values as exact values whenever suitable.*
☒	☐	For Bayesian analysis, information on the choice of priors and Markov chain Monte Carlo settings
☒	☐	For hierarchical and complex designs, identification of the appropriate level for tests and full reporting of outcomes
☒	☐	Estimates of effect sizes (e.g. Cohen's *d*, Pearson's *r*), indicating how they were calculated

Our web collection on statistics for biologists contains articles on many of the points above.

Software and code

Policy information about availability of computer code

Data collection	All software used to perform data collection are described in the methods section of the manuscript or the supportive information.<br>Multiparametric Flow cytometry data was collected on FACSAriaIII and LSRFortessa X-20 with FACSDiva software version 8.0.1 (BD, Biosciences)<br>Hematoxylin and eosin images were generated using NDP.view2 software version 2.9.29 (Hamamatsu).<br>The immunofluoresce images were acquired and generated using Zen software version 3.1 (Carl Zeiss) and LAS AF Lite software version 4.0 (Leica), respectively.
Data analysis	Statistical significance between two groups was tested using the nonparametric unpaired Mann-Whitney U test. Nonparametric Spearman test was used to calculate the correlation between two variables, presented as r value. Data are presented as median (IQR). Differences were considered significant at p<0.05. GraphPad Prism version 9 was used for most analyses (GraphPad Software). Data presented are from at least three independent experiments.<br>For bulk RNA-seq analysis, sequencing data were uploaded on the galaxy web platform and the public sever https://usegalaxy.eu was used for mapping and counting analysis. Reads were trimmed for adapter sequences using Trimmomatic software (version 0.38.1) and aligned to the Hg19 reference genome using HiSat2 software (version 2.2.1). The expression count matrix was generated using HTSeq (version 0.9.1) using the Ensembl genome annotation GRCh38.p13. Normalization and differential expression analyses were performed with the website iDEP version 0.93 (http://bioinformatics.sdstate.edu/idep/).<br>For single-cell bioinformatic analysis, raw sequencing data was aligned, annotated, demultiplexed and filtered using Cell Ranger Software (v.6.0.1) with a custom-built reference (reference genome GRCh38 and gene annotation Ensembl 98 with EGFP sequence included). Downstream analyses were done using R 4.1.0. Raw UMI counts were loaded and the "background soup" was removed using SoupX (version 1.5.2). Decontaminated UMIs were then filtered to discard any doublet using DoubletFinder (version 2.0.3). Scaling and normalization were performed using Seurat (version 4.0.3). Pseudotime analysis was performed using Monocle3 (version 1.0.0). Receptor-ligand interaction analysis was done with CellPhoneDB (version 2.1.4). Integration of D45, D58 and the public human cell landscape dataset were done using Seurat's reciprocal PCA integration with 30 dimensions, following Seurat's guidelines.

For manuscripts utilizing custom algorithms or software that are central to the research but not yet described in published literature, software must be made available to editors and reviewers. We strongly encourage code deposition in a community repository (e.g. GitHub). See the Nature Portfolio guidelines for submitting code & software for further information.

Data

Policy information about availability of data

All manuscripts must include a data availability statement. This statement should provide the following information, where applicable:

- Accession codes, unique identifiers, or web links for publicly available datasets
- A description of any restrictions on data availability
- For clinical datasets or third party data, please ensure that the statement adheres to our policy

Bulk RNA-seq and Single-cell RNA-seq data have been deposited in the NCBI Gene Expression Omnibus under accession number GSE181452 and GSE203085, respectively. In addition, data from bulk RNA-seq control samples (cAMP condition from day 37 and 45) have been deposited under GSE201558 accession number. scRNAseq data from human thyroid tissue was obtained from Human Cell Landscape (available at https://db.cngb.org/HCL/). The custom-built reference was based on the human reference genome GRCh38 (GenBank accession code GCA_000001405. 15; RefSeq accession code GCF_000001405). Source data are provided with this paper.

Field-specific reporting

Please select the one below that is the best fit for your research. If you are not sure, read the appropriate sections before making your selection.

☒ Life sciences ☐ Behavioural & social sciences ☐ Ecological, evolutionary & environmental sciences

For a reference copy of the document with all sections, see nature.com/documents/nr-reporting-summary-flat.pdf

Life sciences study design

All studies must disclose on these points even when the disclosure is negative.

Sample size	Sample-size was not calculated and the size was chosen according to our previous study in mESC-derived thyroid (Antonica, et al, Nature, 2012) where 20 mice were used. For this study we increased the number to 22 animals, since NOD-SCID animals are already known to be sensitive to radiation which combined to the transplantation could lead to a sample-size reduction during the experiment.
Data exclusions	No data was excluded.
Replication	For all the organoids quantification, analysis were performed at least 6 times for qPCR and at least 3 times for Flow cytometry. For bulk RNA-sequencing analysis, 2 times 4 wells (matrigel drops containing organoids) were collected together and proceeded for analysis. For single cell RNA-sequencing 12 wells (each time point) were collected together and NKX2-1/GFP+ and NKX2-1/GFP- populations were sorted and sequenced. All the attemps were successful for the transcriptomics analysis and flow cytometry. Differentiation replication rate was higher than 90% (at least 10 distinct experiments), and is strongly dependent of the use of fresh medium. Immunfluorescence stainings were performed every differentiation (at least 10 times). In vivo studies were performed 3 times, the 2 first ones in order to set the protocol, with failure in one case when thyroid follicles were extensively purified and stromal cells quite completely depleted. The experiment described in the study shows success of the graft and thyroid tissue generation in 10 out of 10 mice.
Randomization	For in vitro experiments, all the conditions were cultured in parallel starting from the same source of hESCs. Treated and untreated samples were chosen among the wells which were showing to be similar macroscopically. Randomization was not performed in our in vivo study, since all the animals at the beginning of the experiment had the same characteristics, such as: age, sex, diet and weight. The choice for RAI injection was aleatory while grafting was dependent of prior RAI-injection.
Blinding	Blinding was not used in our in vivo study design, since the animals irradiated (RAI) and grafted were macroscopically identifiable and the outcome was not dependent of any additional treatment performed by the researchers than the graft. However, all the animals were identified by codes and the blood analysis (T4, T3 and TSH) and liver analysis (Dio1 mRNA) were blinded.

Reporting for specific materials, systems and methods

We require information from authors about some types of materials, experimental systems and methods used in many studies. Here, indicate whether each material, system or method listed is relevant to your study. If you are not sure if a list item applies to your research, read the appropriate section before selecting a response.

Materials & experimental systems

n/a	Involved in the study
☐	☒ Antibodies
☐	☒ Eukaryotic cell lines
☒	☐ Palaeontology and archaeology
☐	☒ Animals and other organisms
☐	☒ Human research participants
☒	☐ Clinical data
☒	☐ Dual use research of concern

Methods

n/a	Involved in the study
☒	☐ ChIP-seq
☐	☒ Flow cytometry
☒	☐ MRI-based neuroimaging

Antibodies

Antibodies used	The following primary antibodies were used: goat anti-AFP (sc-8108 Santa Cruz, 1:100), mouse anti--III Tubulin (MMS-435P-200 Eurogentec, 1:1000), rabbit anti-SMA (ab32575 Abcam, 1:1000), rabbit anti-NKX2-1/TTF1 (ab76013 Abcam, 1:500), rabbit anti-PAX8 (59019 Cell Signaling, 1:500), rabbit anti-TG (A0251 Dako, 1:2,000), mouse anti-TG (Abcam, Ab187378, 1:250), mouse anti-TPO (sc-58432 Santa Cruz, 1:100), goat anti-T4 (orb11479 Biorbyt, 1:1,000), mouse anti-T4 (MA5-14716 Invitrogen, 1:100), mouse anti-E-cadherin (610181 BD, 1:1,000), goat anti-CD31 (AF3628 R&D, 1:100), mouse anti-Human Nuclear Antigen (HNA) (ab190710 Abcam, 1:250), and Phalloidin 647 (10656353 Invitrogen, 1:100), rabbit anti-FOXA2 (Ab40874 Abcam, 1:1,000), goat anti-SOX17 (Sc-17355 Santa Cruz, 1:100), mouse anti-Troponin T (Ab8295 Abcam, 1:200), mouse anti-MUC5AC (Ab3649 Abcam, 1:250), mouse anti-TP63 (Ab735 Abcam, 1:50) and rabbit anti-KRT5 (#25807 Cell Signaling, 1:500). Secondary antibodies were donkey anti-mouse Cy3-conjugated (Jackson Immunoresearch, 715-165-150, 1;500), donkey anti-rabbit Cy3-conjugated (Jackson Immunoresearch, 711-165-152, 1:500), donkey anti-goat cy3- conjugated (Jackson Immunoresearch, 705-165-147, 1;500), donkey anti-mouse IgG Alexa fluor 488-conjugated (Jackson Immunoresearch, 715-545-150, 1:500), donkey anti-mouse IgG Alexa fluor 647-conjugated (Jackson Immunoresearch, 715-605-150, 1:500), donkey anti-rabbit IgG Alexa fluor 647-conjugated (Jackson Immunoresearch, 711-605-152, 1:500), and Hoechst (33342, Invitrogen, 1:1,000).
Validation	Primary antibodies were tested and validated for Immunofluorescence in human samples using the concentrations described above. Briefly, Human thyroid sample embedded in paraffin wassectioned (5 µm), mounted on glass slides, deparaffinized, and rehydrated. Antigen retrieval was performed by incubating the sections for 10 min in the microwave (850 W) in Sodium Citrate Buffer (10 mM Sodium Citrate, 0.05% Tween 20, pH 6.0). After cooling, the sections were rinsed with PBS and then blocked with 1% BSA and 10% horse serum PBS solution for 1 h at RT. Primary antibodies were diluted in the blocking solution and incubated overnight at 4°C. The sections were rinsed three times in PBS and incubated with Hoechst 33342 (Invitrogen) and secondary antibodies diluted in blocking solution for 1 h at room temperature. Slides were mounted with Glycergel (Dako) and imaged using Zeiss Axio Observer Z1 microscope with AxioCamMR3 camera, or a Leica DMI6000 with DFC365FX camera.<br>Primary antibody tested and validated for Flow cytometry in human samples: anti-PAX8 (59019 Cell Signaling, 1:100). The BrdU Flow Kit protocol (BD Biosciences) was used for PAX8 staining.

Eukaryotic cell lines

Policy information about cell lines

Cell line source(s)	The human embryonic stem cell line HES3 used in this study was a gift from Prof Elefanty (Goulburn, A. L. et al. A targeted NKX2.1 human embryonic stem cell reporter line enables identification of human basal forebrain derivatives. Stem Cells 29, 462–473 (2011). Commercial source: ES Cell International Pte Ltd. (ESIBI).
Authentication	The hESC-line was modified and the final hESC-NKX2-1-PAX8 line was registered and approved by the European Human Pluripotent Stem Cell Registry (hPSCreg) as ESIBIe003-A-6. The cell line was registered at hPSCreg following the standard procedure which includes: Documentation of ethical provenance of the cells according to national regulation; Evidence for pluripotency of the cell; That the cell is, in principle, available for research; Protocol and documentation of cell genomic modifications. In addition, were included cultivation protocol, batch information, expression arrays, genetic information, donor phenotype information, details on differentiation potential or usage in specific applications or projects and publications.
Mycoplasma contamination	Mycoplasma contamination was excluded by PCR test (Mycoplasma Detection Testing, Thermo Fisher)
Commonly misidentified lines<br>(See ICLAC register)	No commonly misidentified lines were used in this study.

Animals and other organisms

Policy information about studies involving animals; ARRIVE guidelines recommended for reporting animal research

Laboratory animals	For the in vivo studies we used five-week-old female non-obese and non-diabetic mice with severe combined immunodeficiency (NOD -SCID). The housing conditions of all animals were strictly following the ethical regulations. The room temperature ranged from 20 and 25°C. The relative ambient humidity at the level of mouse cages was 55% +/-15. Each cage was provided with food, water and two types of nesting material. Semi-natural light cycle of 12:12 was used.
Wild animals	The study not involve wild animals

Field-collected samples	No field collected samples were used in the study
Ethics oversight	All animal experiments were performed in accordance with local Animal Ethics (Commission d'Ethique du Bien-Être Animal (CEBEA) Faculté de Médecine ULB, Project CMMI-2020-01).

Note that full information on the approval of the study protocol must also be provided in the manuscript.

Human research participants

Policy information about studies involving human research participants

Population characteristics	Human thyroid tissue histologically normal was obtained from a patient undergoing thyroidectomy.
Recruitment	The recruitment was based on the patient agreement and availability of the tissue.
Ethics oversight	Hopital Erasme-ULB Ethics Committee approval; P2016/260.

Note that full information on the approval of the study protocol must also be provided in the manuscript.

Flow Cytometry

Plots

Confirm that:

[x] The axis labels state the marker and fluorochrome used (e.g. CD4-FITC).

[x] The axis scales are clearly visible. Include numbers along axes only for bottom left plot of group (a 'group' is an analysis of identical markers).

[x] All plots are contour plots with outliers or pseudocolor plots.

[x] A numerical value for number of cells or percentage (with statistics) is provided.

Methodology

Sample preparation	hESCs under thyroid differentiation protocol were incubated with 10 µL/ml of 1mM BrdU in culture medium for three hours, at several time points. Then treated cells and controls were washed once with PBS, collected and prepared for flow cytometry immunostaining as follows: Matrigel drops (at least 4 samples per time point) were first digested with HBSS solution containing 10 U/ml dispase II (Roche) and 125 U/ml collagenase type IV (Gibco, Thermo Fisher) for 30-60 min at 37° C; then a single cell suspension was obtained by dissociation with TripLE Express (Thermo Fisher) for 10-15 min incubation at 37°C, the enzymes were inactivated by addition of differentiation medium. After centrifugation, samples were rinsed with PBS, fixed and stained following the APC BrdU Flow Kit protocol (BD Biosciences). In addition, PAX8 antibody was used to stain the thyroid cells. NKX2-1GFP, PAX8 and BrdU incorporated cells (BrdU+) data were obtained and processed using an LSR-Fortessa X-20 flow cytometer and FACSDiva software (BD Biosciences). Unstained cells and isotype controls were included in all experiments. In addition, the percentage of NKX2-1GFP cells was used to estimate the thyroid generation efficiency of our protocol. Also, NKX2-1GFP/PAX8 cells were used to evaluate the thyroid fate commitment. Gate strategies are exemplified at Supplementary Fig. 1h-i.
Instrument	LSR-Fortessa X-20 flow cytometer
Software	FACSDiva software (BD Biosciences)
Cell population abundance	Flow cytometry analysis revealed that the % of NKX2-1-GFP+ cells vary from 10% (day 16) to 25% (end of the protocol). The cell population was determined using endogenous NKX2-1-GFP expression (FITC) while viable cells where selected using Calcein Violet AM dye (405 nm). To determine the proliferation rate among NKX2-1-GFP+ population, cells where stained with KI67 antibody and secondary anti-rabbit cy5 (see above and methods section). Overtime analysis shows that from day 16 to day 38, more than 90% of the NKX2-1-GFP+ cells were KI67+, while the percentage reduced to 40% at day 47.
Gating strategy	For NKX2-1-GFP+ cell population assessment: Initially cell population was selected using SSC vs FSC density plot. A gate has been applied to identify the single cells and to remove debris. Additionally, singlets were selected using FSC-H and FSC-A gate strategy. After, a gate was applied to select viable cells using BV421-A and FSC-A. Finally, NKX2-1-GFP+ population was selected using FITC-A and FSC-A. NKX2-1-GFP- cells (undifferentiated cells), were used as negative controls for GFP gate strategy.<br><br>For NKX2-1-GFP+/PAX8 cell population assessment: Initially, viable single cell population and NKX2-1-GFP+ cells were selected as described above. Then, PAX8+ cell population was determined using APC-A and FSC-A. Finally, NKX2-1-GFP+/PAX8 + cell population was obtained using FITC-A and APC-A plot. Unstained and stained only with the secondary antibody (Rabbit cy5) samples were used as negative controls for the PAX8 gate strategy.<br><br>For NKX2-1GFP/BrdU cell population assessment was performed following the APC BrdU Flow Kit protocol (BD Biosciences) kit instructions.

[x] Tick this box to confirm that a figure exemplifying the gating strategy is provided in the Supplementary Information.
